# Supplementary material for: Multiple episodes of ice loss from the Wilkes Subglacial Basin during the Last Interglacial
Source: Nat Commun. 2023 Apr 18;14:2129. doi: 10.1038/s41467-023-37325-y (PMC10113383; doi:10.1038/s41467-023-37325-y)
Supplement: Supplementary file 1 — Supplementary Information [file 41467_2023_37325_MOESM1_ESM.pdf]

Supplementary Information for

Multiple episodes of ice loss from the Wilkes Subglacial Basin during the Last Interglacial

Authors:

Mutsumi Iizuka[1,2,3*], Osamu Seki[1*], David J. Wilson[4], Yusuke Suganuma[5,6], Keiji Horikawa[7], Tina van de Flierdt[8,9], Minoru Ikehara[10], Takuya Itaki[3], Tomohisa Irino[2], Masanobu Yamamoto[2], Motohiro Hirabayashi[5], Hiroyuki Matsuzaki[11] Saiko Sugisaki[2]

Affiliations:

1. Institute of Low Temperature Science, Hokkaido University, Sapporo, Japan

2. Graduate School of Environmental Science, Hokkaido University, Sapporo, Japan

3. Institute of Geology and Geoinformation, The National Institute of Advanced Industrial Science and Technology (AIST), Tsukuba, Japan

4. London Geochemistry and Isotope Centre (LOGIC), Institute of Earth and Planetary Sciences, University College London and Birkbeck, University of London, UK

5. National Institute of Polar Research, Tachikawa, Japan

6. Department of Polar Science, School of Multidisciplinary Sciences, The Graduate University for Advanced Studies (SOKENDAI), Tachikawa, Japan

7. Faculty of Science, Academic Assembly, University of Toyama, Gofuku, Japan

8. Department of Earth Science and Engineering, Imperial College London, London, UK

9. Grantham Institute-Climate Change and the Environment, Imperial College London, London, UK

10. Center for Advanced Marine Core Research, Kochi University, Nankoku, Japan

11. Micro Analysis Laboratory, Tandem accelerator (MALT), The University of Tokyo, Bunkyo, Japan

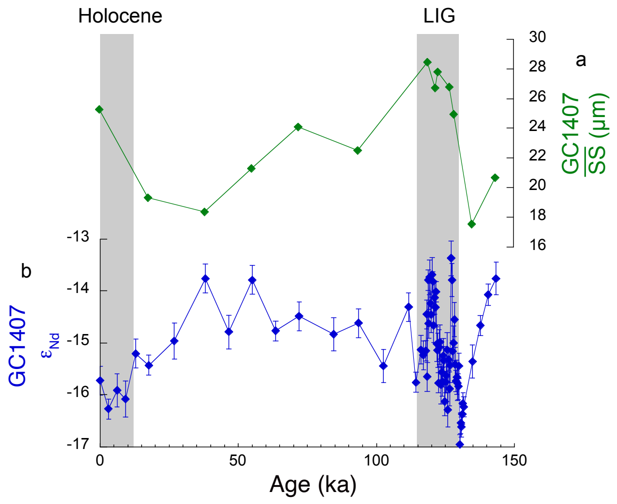

Supplementary Figure 1 | Grain-size analysis in core GC1407 from the Last Interglacial to the Holocene a, Mean grain size of the sortable silt fraction ($\overline{SS}$: 10–63 µm) in core GC1407 (green diamonds). b, Detrital sediment Nd isotopes (ε_{Nd}) in core GC1407 (blue diamonds) (error bars are 2 s.d.). Grey bars highlight the Holocene (11–0 ka) and Last Interglacial (LIG; 130–115 ka) intervals.

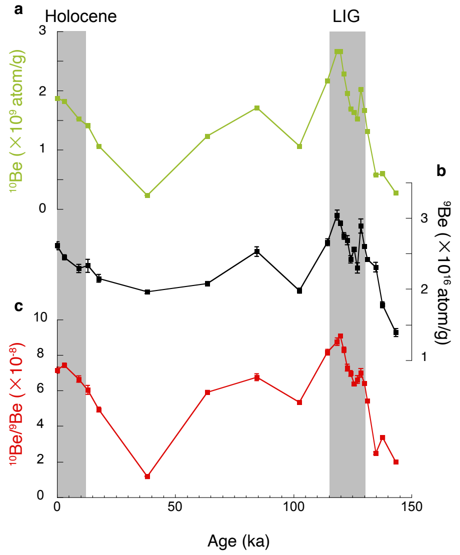

Supplementary Figure 2 | Authigenic Be isotope records in core GC1407 from the Last Interglacial to the Holocene. a, ^{10}Be record (light green squares). b, ^{9}Be record (black squares). c, $^{10}Be/^{9}Be$ record (red squares) (error bars are 1 s.d.). Grey bars highlight the Holocene (11–0 ka) and Last Interglacial (LIG; 130–115 ka) intervals.

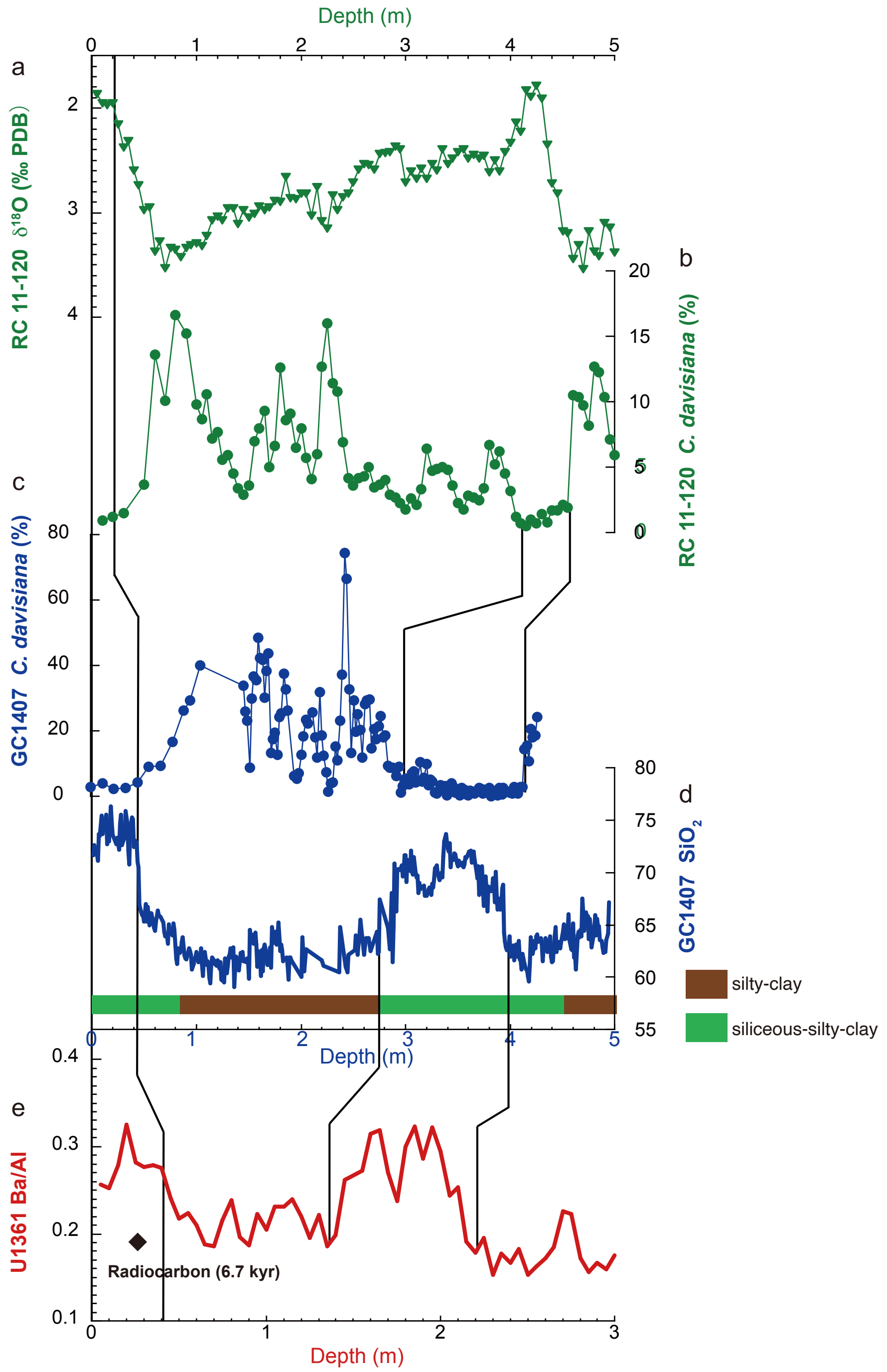

Supplementary Figure 3 | Chronological constraints from the Last Interglacial to the Holocene. a and b, Planktonic foraminifera (*Globigerina bulloides*) $\delta^{18}O$ and *C. davisiana* abundance in core RC11-120[78] (green triangles and dots). c and d, *C. davisiana* abundance and SiO_2 content with lithological log in core GC1407 (blue dots and line). e, Ba/Al ratios (from XRF core-scanning) in core U1361A (red line), and radiocarbon age indicated by black diamond[17]. Vertical lines indicate tie points.

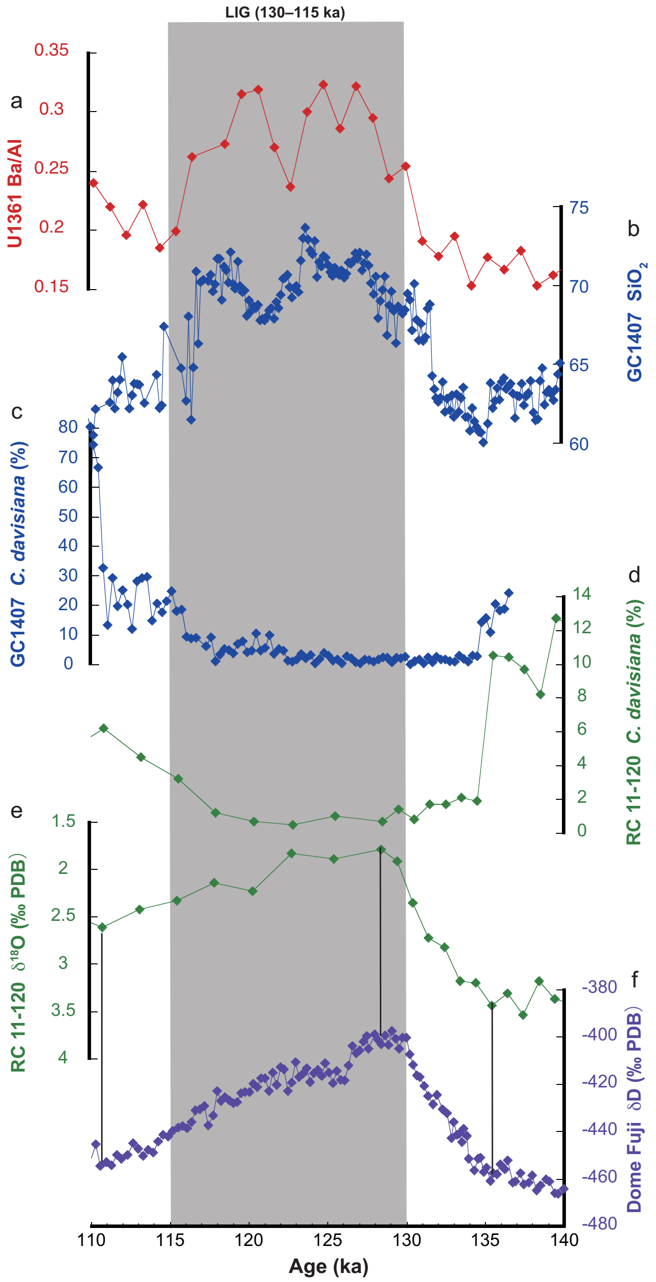

Supplementary Figure 4 | Transferring marine sediment cores onto the AICC2012 chronology during the Last Interglacial. a, Ba/Al ratios (from XRF core-scanning) in core U1361A[17] (red symbols and line). b and c, SiO_2 content and *C. davisiana* abundance in core GC1407 (blue symbols and lines). d and e, *C. davisiana* abundance and planktonic foraminiferal $\delta^{18}O$ in core RC11-120[76,77,78] (green symbols and lines). f, δD in Dome Fuji ice core[80] (purple symbols and line). Vertical lines indicate tie points. Grey bar highlights the Last Interglacial (LIG; 130–115 ka).

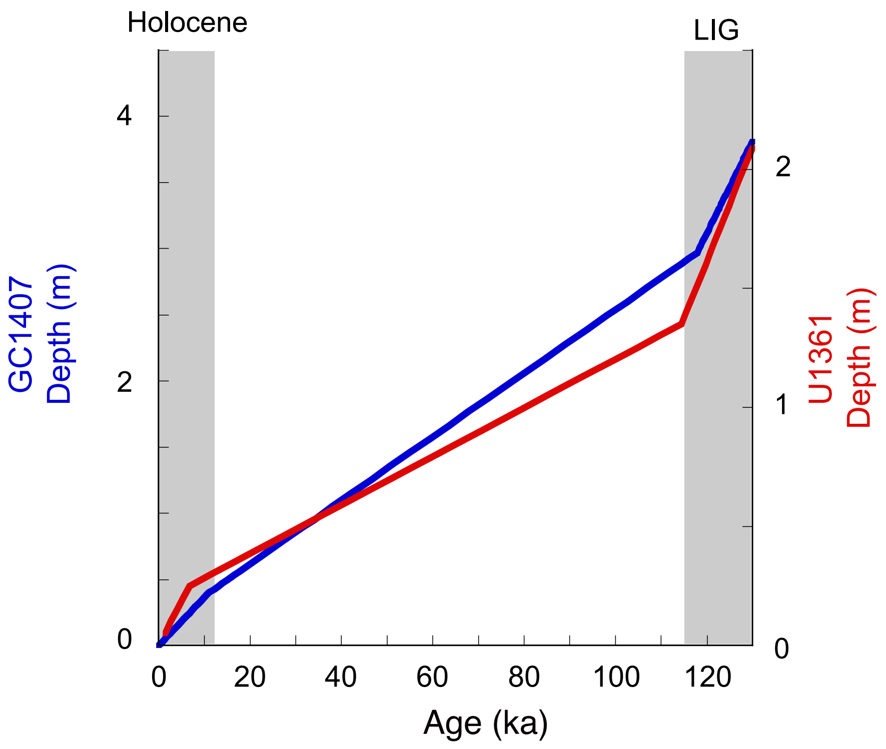

Supplementary Figure 5 | Depth-age models in cores GC1407 and U1361A. Depth versus age plot showing sedimentation rates in cores GC1407 (blue line) and U1361A (red line). Grey bars highlight the Holocene (11–0 ka) and Last Interglacial (LIG; 130–115 ka) intervals.
