# Supplementary material for: Multiple episodes of ice loss from the Wilkes Subglacial Basin during the Last Interglacial
Source: Nat Commun. 2023 Apr 18;14:2129. doi: 10.1038/s41467-023-37325-y (PMC10113383; doi:10.1038/s41467-023-37325-y)
Supplement: Supplementary file 3 — Description of Additional Supplementary Files [file 41467_2023_37325_MOESM3_ESM.pdf]

Description of Additional Supplementary Files:

Supplementary Datasets 1-6: The supplementary file contains the following data tables for GC1407.

Supplementary Dataset 1: Chronostratigraphic constraints.

Supplementary Dataset 2: Detrital sediment (<63 µm fraction) Nd isotope measurements.

Supplementary Dataset 3: Detrital sediment Be isotpoe measurements.

Supplementary Dataset 4: Grain size measurements.

Supplementary Dataset 5: C.davisiana relative abundance counts.

Supplementary Dataset 6: SiO2 by XRF scanning.

Supplementary Dataset 1: GC1407

Supplementary Dataset 2: Chronostratigraphic constraints

Supplementary Dataset 3: Detrital sediment (<63 µm fraction) Nd isotope measurements

Supplementary Dataset 4: Detrital sediment Be isotpoe measurements

Grain size measurements

Supplementary Dataset 5: C.davisiana relative abundance counts

Supplementary Dataset 6. SiO2 by XRF scanning.
